# Acne management in Norway: GP and dermatologist prescriptions (2012–2019): a nationwide overview

**DOI:** 10.3399/BJGPO.2024.0211

**Published:** 2025-09-24

**Authors:** Cathrine S Christiansen, Sigurd Høye, Morten Lindbaek, Jon Anders Halvorsen, Louise Emilsson

**Affiliations:** 1 The Antibiotic Centre for Primary Care, Department of General Practice, Institute of Health and Society, University of Oslo, Oslo, Norway; 2 Department of General Practice, Institute of Health and Society, University of Oslo, Oslo, Norway; 3 Department of Dermatology, Institute of Clinical Medicine, Oslo University Hospital, Oslo, Norway; 4 Vårdcentralen Nysäter and Centre for Clinical Research, County Council of Värmland, Värmland, Sweden; 5 General Practice Research Unit (AFE), Department of General Practice, Institute of Health and Society, University of Oslo, Oslo, Norway; 6 Department of Medical Epidemiology and Biostatistics, Karolinska Institute, Stockholm, Sweden

**Keywords:** dermatology, acne vulgaris, general practice

## Abstract

**Background:**

Acne is common and associated with negative psychosocial health and risk of permanent skin alterations. GPs prescribe the main portion of antibiotics used for acne. Increased isotretinoin prescription by GPs can potentially reduce overall antibiotic use, but prescription practice and trends are unknown.

**Aim:**

To examine acne treatment in Norway and quantify prescription and initiation of isotretinoin and tetracyclines.

**Design & setting:**

An observational study linking data from health registries. Data were collected from the Norwegian Prescription Database (NorPD), the national GP claims register (KUHR database), and the Regular General Practitioner (RGP) registry.

**Method:**

All patients aged 12–39 years who received an acne diagnosis or were prescribed acne medication in Norway 2012–2019 were included. Linear regression was used to explore time trends.

**Results:**

In total, 316 075 patients were included (63% female). Yearly prevalence of systemic treatment increased from 1.9 in 2012 to 2.4% in 2019; isotretinoin increased by +123%, tetracyclines by +4% as measured in defined daily doses (DDDs). Topical treatment increased by +13% as measured by number of prescriptions. GP prescription of tetracyclines decreased 11%; however, courses had a mean duration of 160 days, which is longer than the recommended 90 days, and only 26% had a co-occurring topical treatment prescription. GPs initiated 5% of isotretinoin courses in 2012, versus 10% in 2019, and 19% (*n* = 1339) of GPs initiated isotretinoin at least once during the study period.

**Conclusion:**

GPs reduced their prescription of tetracyclines, but our data still show potential for further improvements in prescribing practice. Increased isotretinoin prescription by GPs may lead to reduced antibiotic use and better treatment regimens for moderate-to-severe acne.

## How this fits in

In patients with moderate-to-severe acne, early treatment with isotretinoin is important to reduce psychosocial burden and irreversible skin alterations. Antimicrobial resistance (AMR) is a growing global concern and treatment with tetracycline should be used wisely. This study shows that Norwegian GPs have increased their prescription of isotretinoin and reduced prescription of tetracyclines. It is important to further acknowledge this as good practice and encourage GPs to initiate isotretinoin treatment safely, to contribute to reducing AMR and providing optimal acne care.

## Introduction

Acne vulgaris (acne) is common among youths and is mainly treated in general practice. Approximately 85% of adolescents experience acne, with 15–20% facing moderate-to-severe cases, and it might also appear or extend into adulthood.^1–3^ Acne’s psychosocial burden is well documented and youths with acne report more anxiety, depression, and suicidal thoughts than their peers.^1,4^ The presence of psychological symptoms should initiate a more proactive treatment approach.^5^ Further, acne can give permanent scarring and post-inflammatory hyperpigmentation, with early treatment being the only preventive measure.^6–8^

Medical acne treatment is based on the following four main alternatives: topical treatment; systemic antibiotics; hormonal therapy for women; and systemic retinoids. Guidelines^9^ recommend topical retinoids and/or benzoyl peroxide for all severities of acne and mandate their use alongside systemic antibiotics.^10–12^ Monotherapy with topical or systemic antibiotics should be avoided.^8^ Systemic antibiotics for moderate-to-severe acne have been used for decades, predominantly tetracyclines, which are both antibacterial and anti-inflammatory.^12^ Use should be discontinued after 3 months,^9,11,13^ but are often prescribed longer.^13–16^ The prescription of systemic tetracyclines for acne increased in Norway from 2005–2015.^17^ Increased use of systemic tetracyclines and longer duration of treatment than recommended contributes to antimicrobial resistance.^18,19^ The majority of tetracyclines for acne treatment are prescribed by GPs.^17^

Isotretinoin targets all main mechanisms in the acne pathogenesis^20^ and is indicated for severe acne (for example, nodular acne or acne conglobata or acne at risk of permanent scarring) or acne resistant to adequate standard treatment with systemic antibiotics and topical treatment. Side effects are common but usually manageable with symptomatic treatment.^21,22^ It is also highly teratogenic, and contraceptive control is required in female patients. Isotretinoin treatment has further been associated with depression and suicidal thoughts, but this association remains controversial^23^ as psychological symptoms may reflect the burden of acne itself, rather than an adverse effect of isotretinoin, and successful treatment with isotretinoin has also been shown to reduce symptoms of depression.^24,25^ Nevertheless, it is recommended to monitor isotretinoin patients for psychiatric symptoms.^22,26^ GPs are experienced in both contraceptive care and psychiatric follow-up, making them well-positioned to prescribe isotretinoin.

In many countries isotretinoin prescription is only approved for dermatologists while GP prescription is encouraged in some countries. In the Netherlands, GPs are guided to prescribe isotretinoin by national guidelines.^27^ In New Zealand GPs have prescribed isotretinoin since 2009^28^ and in 2012 58% of issued prescriptions were by GPs.^29^ An Irish survey showed that 17% of GPs initiate isotretinoin, but also identified suboptimal adherence to recommended monitoring.^30^ In Norway, patients need a GP referral to see a dermatologist, except for a few private dermatologists not covered by government subsidies, limiting access to these services.

There is currently no clear consensus whether isotretinoin should be initiated sooner to reduce patient suffering and to lower the use of systemic tetracyclines, potentially even as a first-line treatment.^17,20^ In Norway, GPs have been allowed to prescribe isotretinoin since 2007, but the extent of initiation and prescribing practice is unknown. This study aimed to present prescription patterns for acne therapy in Norway.

## Method

We conducted an observational study linking data from nationwide health registries in Norway in the period from 2012–2019. Individual-level data were collected from several Norwegian nationwide health registries: the Norwegian Prescription Database (NorPD), the national GP claims register (KUHR database), and the Regular General Practitioner (RGP) registry. The data sources were linked by the unique personal identification number assigned to every resident of Norway, so individuals can be followed over time and across registers and databases. Population statistics were obtained from Statistics Norway.

The NorPD contains data on all issued drugs to patients in ambulatory care and covers the entire Norwegian population. We acquired data on the following code prescriptions in the Anatomical Therapeutic Chemical Classification (ATC) system: D10A* (benzoyl peroxide, azelaic acid, topical antibacterials, and topical retinoids), D10B* (isotretinoin), and J01A* oral antibiotics. Systemic antibiotics used to treat acne included in this study were doxycycline (J01AA02), lymecycline (J01AA04), and tetracycline (J01AA07). For doxycycline, we only included prescriptions of 50 defined daily dose (DDDs) or more, to exclude courses of shorter duration most likely prescribed for respiratory or sexually transmitted infections. The recommended prescription of tetracyclines for acne is ½ DDD daily for 3 months, that is, the usual prescription would be 50 DDD.

The KUHR database is administrated by HELFO (The Norwegian Health Economics Administration), which receives reimbursement claims from all GPs, out-of-hours (OOH) service doctors, and private specialists with public contract. We obtained all claims for consultations registered with the International Classification of Primary Care (ICPC)-2 code ’S96 Acne’ or International Classification of Diseases (ICD)-10 code ’L70 Acne’.

The RGP registry contains information about all regular GPs (RGPs) and their listed patients. The RGP’s sex, age, and centrality were recorded at their first occurrence in the data.

### Population

We included patients aged 12–39 years, who had received either an acne diagnosis or a dispensed medication (topical treatment, systemic tetracyclines, or isotretinoin) for acne before the age of 40 years. The data for each patient was excluded from the year they turned 40 years. Prevalence was defined as number of patients per 100 inhabitants of same age and sex. In a post-hoc analysis to explore age (12–18 years, 19–25 years, 26-32 years, and 33–39 years) and sex (male, female) treatment allocation differences, we present average yearly prevalence of the treatments calculated as total number of individuals with the relevant prescription divided by the total number of Norwegian inhabitants in the relevant age and sex categories. We applied a washout period of 6 months without dispensed isotretinoin, to make sure the treatment was not initiated by another specialty group, when defining whether isotretinoin treatment was initiated by a GP (versus treatment continued by a GP but initiated by a dermatologist). Specialty areas were defined as follows: ’Dermatology’ encompassed all registered specialists in dermatology; ’General practitioners (GPs)’ were registered specialists in general practice, and physicians where the contact in the KUHR-database was registered as general practice. This also included physicians with other specialties that worked in general practice, except for specialists in dermatology who were always defined as ’Dermatologists’. ’Other medical doctor’ were physicians in other specialties or physicians without specialty training. A treatment course was defined as prescriptions of systemic treatment, dispensed less than 365 days apart. If a drug was dispensed 366 days after the previous, it was defined as a new treatment course. Dispensed topical treatment within 90 days before an episode of tetracycline treatment was defined as concurrent medication.

### Statistical analysis

Descriptive statistics are presented including mean, standard deviation (SD), range, and 95% confidence intervals (CIs). Linear regression analysis was used to explore time trends. All statistical analyses were performed using Stata/MP (version 16.1).

## Results

**Figure 1.**
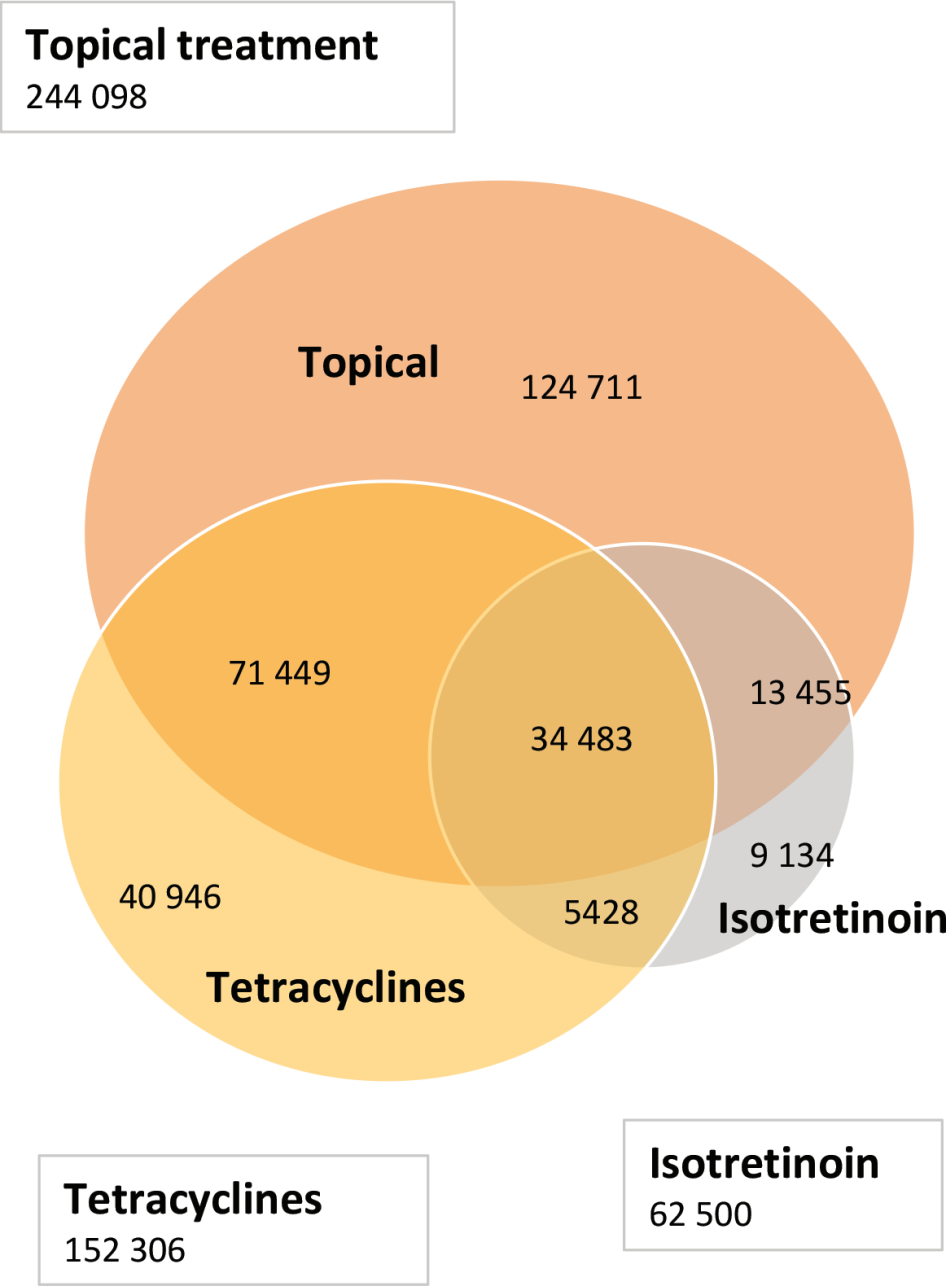
Number of patients and combinations of treatment from 2012–2019

In total, 316 075 patients with acne were included, 63% were female, and mean age at first registration was 22.8 years. A total of 299 606 (95%) received at least one acne prescription and 186 777 (59 %) had an acne diagnosis registered (Table 1). Topical treatment was prescribed to 244 098 (77%) patients, tetracycline to 152 306 (48%), and isotretinoin to 62 500 (20%) (Figure 1). Yearly isotretinoin prescription increased by +123% (which corresponded to a yearly increase of 217 048 DDDs; *P*<0.000, 95% confidence interval [CI] = 201 583 to 232 513), and tetracyclines +4% as measured in DDDs during the study period (Table 2). Topical treatment increased by +13% measured in number of prescriptions. Of all patients who received isotretinoin, 51 808 (83%) had an acne diagnosis registered during the study period. Among the identified 7085 GPs, 3601 (51%) prescribed isotretinoin, and 1339 (19%) initiated isotretinoin treatment at least once during the study period.

**Table 1.** Number of patients and combinations of treatments

	Total		Female		Male	
**Patient characteristics**	Number	Proportion	Number	Proportion	Number	Proportion
Total	316 075		200 143	63%	115 932	37%
Acne diagnosis	186 777	59%	114 394	57%	72 383	62%
Received any kind of treatment	299 606	95%	190 191	95%	109 415	94%
Patient with acne diagnosis who received any kind of treatment	170 308	91%	104 442	91%	65 866	91%
Patient with acne diagnosis with no prescribed treatment	16 469	9%	9952	9%	6517	9%
**Type and combination of treatments**						
Topical treatment	244 098	77%	160 721	80%	83 377	72%
Systemic tetracyclines	152 306	48%	92 158	46%	60 148	52%
Isotretinoin	62 500	20%	34 185	17%	28 315	24%
Topical treatment only	124 711	39%	85 882	43%	38 829	33%
Topical treatment and systemic tetracycline only	71 449	23%	46 861	23%	24 588	21%
Topical treatment and isotretinoin only	13 455	4%	8105	4%	5350	5%
Topical treatment, systemic tetracycline, and isotretinoin	34 483	11%	19 873	10%	14 610	13%
Systemic tetracyclines only	40 946	13%	23 263	12%	17 683	15%
Systemic tetracyclines and isotretinoin only	5428	2%	2161	1%	3267	3%
Systemic isotretinoin only	9134	3%	4046	2%	5088	4%

**Table 2.** Defined daily dosage prescribed for isotretinoin and tetracycline per specialty and year

**Tetracycline**	**Total**	**GP**		**Dermatologist**		**Other medical doctor**	
	**DDDs**	**DDDs**	**Proportion**	**DDDs**	**Proportion**	**DDDs**	**Proportion**
2012	1 802 195	1 185 979	66%	501 182	28%	115 035	6%
2013	1 836 953	1 203 300	66%	518 339	28%	115 314	6%
2014	1 952 184	1 262 672	65%	571 510	29%	118 002	6%
2015	1 878 476	1 205 479	64%	551 146	29%	121 851	6%
2016	1 794 496	1 117 678	62%	547 403	31%	129 416	7%
2017	1 785 780	1 079 896	60%	558 828	31%	147 056	8%
2018	1 791 511	1 059 327	59%	562 052	31%	170 132	9%
2019	1 870 767	1 060 176	57%	595 641	32%	214 950	11%
**Change**	4%	-11%		19%		87%	
***P* value (time trend**)	0.70	0.009		0.012		0.004	
**Average yearly change**	-3933	-26 626		9976		12 717	
**95% confidence interval**	-27 389 to 19 522	-43 721 to -9531		3121 to 16 832		5956 to 19 478	
**Isotretinoin**	**Total**	**GP**		**Dermatologist**		**Other medical doctor**	
	**DDDs**	**DDDs**	**Proportion**	**DDDs**	**Proportion**	**DDDs**	**Proportion**
2012	1 256 888	55 278	4%	1 178 259	94%	23 352	2%
2013	1 450 901	85 453	6%	1 342 838	93%	22 610	2%
2014	1 646 300	97 340	6%	1 523 006	93%	25 953	2%
2015	1 870 480	130 450	7%	1 702 940	91%	37 090	2%
2016	2 056 011	188 446	9%	1 808 961	88%	58 604	3%
2017	2 250 146	237 716	11%	1 929 749	86%	82 681	4%
2018	2 529 228	236 231	9%	2 173 734	86%	119 263	5%
2019	2 805 938	272 789	10%	2 364 571	84%	168 578	6%
**Change**	123%	393%		101%		622%	
***P* value (time trend**)	0.000	0.000		0.000		0.001	
**Average yearly change**	217 048	32 804		164 106		20 137	
**95% confidence interval**	201 583 to 232 513	26 962 to 38 647		150 083 to 178 130		11 997 to 28 277	

DDD = defined daily dosage.

### Prevalence

**Figure 2.**
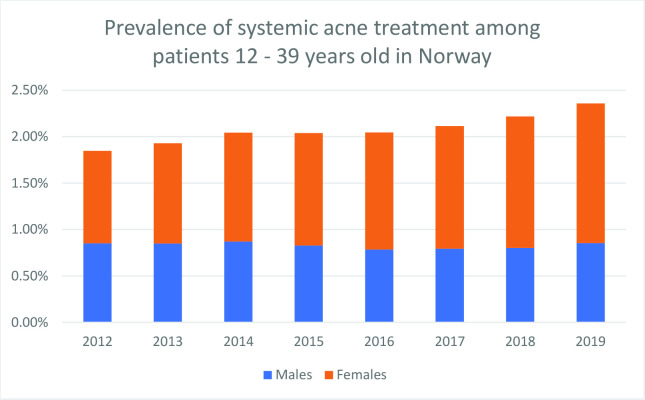
Prevalence of systemic acne treatment among patients aged 12–39 years in Norway

**Figure 3.**
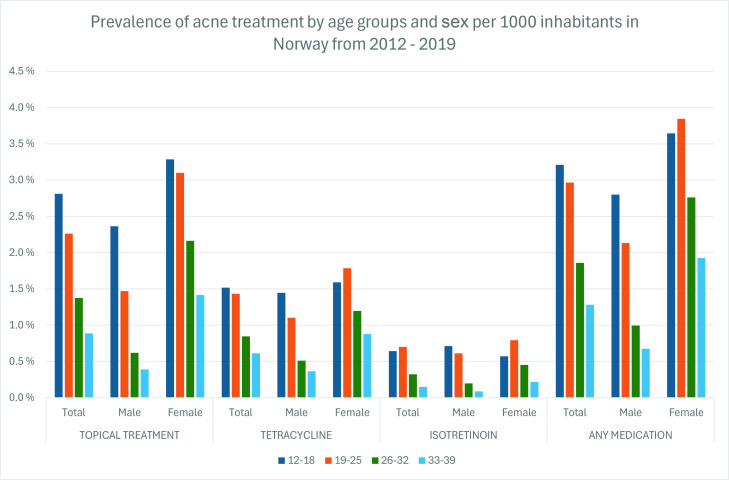
Prevalence of acne treatment by age groups and sex per 1000 inhabitants in Norway from 2012–2019

The prevalence of patients who received any acne treatment was 3.4% in 2012, increasing to 4.3% in 2019; in females, the increase was from 4.3% to 6.0%; in males, it remained stable at around 2.7%. Systemic treatment use (systemic tetracycline and/or isotretinoin) increased from 1.9% in 2012 to 2.4% in 2019; the increase was only significant in females (Figure 2). Age and sex-specific prevalence showed an inverse age-treatment relationship in all specified subgroups except that isotretinoin and tetracycline treatment in females aged 12–18 years were less common than in females aged 19–25 years (Figure 3).

### Tetracycline

The prevalence of tetracyclines use was similar in 2012 (1.54% of the population) versus 2019 (1.56%), showing opposite trends in males and females: a significant increase in females (+18%, *P*<0.01) and significant decrease in males (-20%, *P*<0.001) (Table 3). In 2012, 66% of tetracyclines were prescribed from GPs, 28% from dermatologists, and 6% from other medical doctors versus 57%, 32%, and 11% in 2019. GPs prescription of tetracyclines significantly decreased, and measured in DDDs the yearly average reduction was -26 626 DDDs, 95% CI = -43 721 to -9531, *P*<0.009 (Table 2).

**Table 3.** Prevalence of tetracycline and isotretinoin treatment per 100 000 inhabitants aged 12–39 years

**Tetracyclines**	Total	Males	Females
2012	1543	691	853
2013	1573	667	906
2014	1636	672	964
2015	1559	615	944
2016	1485	559	926
2017	1491	548	943
2018	1505	536	969
2019	1558	549	1009
**Change 2012–2019**	1%	-20%	18%
***P* values**	0.28	0.001	0.010
**Average yearly change**	-9	-25	16
**95% confidence interval**	-27 to 10	-34 to -16	5 to 26
**Isotretinoin**	Total	Males	Females
2012	392	212	180
2013	458	239	219
2014	526	259	267
2015	611	275	336
2016	695	286	409
2017	766	308	458
2018	861	329	532
2019	951	370	581
**Change 2012–2019**	143%	75%	223%
***P* values**	0.00	0.00	0.00
**Average yearly change**	80	20	60
**95% confidence interval**	76 to 84	17 to 24	56 to 64

In total 17% (25 990) of patients on tetracyclines received more than one prescription within 365 days, and 3% (4374) received three or more prescriptions. The average DDDs for tetracycline prescriptions was 80.2, with a median of 50 DDDs, corresponding to an average treatment duration of 160 days (5.3 months). Some 25% of the courses were shorter than 50 DDDs, 38% were exactly 50 DDDs, and 37% were longer than 50 DDDs. Moreover, approximately 16% comprised of more than 100 DDDs, equating to a treatment period of more than 200 days (or around 6.7 months). Of the tetracycline prescriptions, 55% was for lymecycline, 43% for tetracycline, and 2% for doxycycline. Only 26% (48 068) of tetracycline prescriptions were accompanied by a simultaneously (±90 days) prescribed topical treatment, 35% when tetracyclines were prescribed by dermatologists, 23% when prescribed by GPs, and 22% when prescribed by other medical doctors.

### Isotretinoin

Prescription of isotretinoin increased from 0.39% (2012) to 0.95% (2019) of the population (*P*<0.000). The increase was more pronounced in females (from 0.18% to 0.58%, *P*<0.00) than in males (from 0.21% to 0.37%, *P*<0.00), though significant in both groups (Table 3). In 2012 94% of issued DDDs of isotretinoin were prescribed by dermatologists, 4% by GPs, and 2% other medical doctors versus 84%, 10%, and 6% in 2019 (Table 2). GPs quadrupled their yearly issued DDDs of isotretinoin during the study period with an average yearly increase of 32 804 DDDs (95% CI = 26 962 to 38 647, *P*<0.000) and dermatologists doubled theirs with an average yearly increase of 164 106 DDDs (95% CI = 150 083 to 178 130, *P*<0.000) (Table 2). Among 58 244 patients who received isotretinoin, 9423 got at least one prescription by the GP, and 4803 patients (8%) had the prescription initiated by a GP. Initiation by GPs was 5% in 2012 versus 10% in 2019.

More than one treatment course with isotretinoin was received by 6699 patients (11%), and 831 patients (1%) received three courses or more (maximum five courses; seven patients). The average length of the episodes defined in DDDs was 226 days (median 240); that is, 7.5 months treatment.

### Topical treatments

The prescription of topical therapy slightly increased from 2.4% in 2012 to 3.0% in 2019. This change was restricted to females (3.2% to 4.3%), while prescription in males was stable at 1.7%. In total 67% of topical treatments were prescribed by GPs during the whole study period.

## Discussion

### Summary

The overall use of acne therapy increased from 2012 to 2019, more so for isotretinoin compared with tetracyclines and topical treatment. GPs increased isotretinoin prescriptions while reducing tetracyclines. One-third of tetracyclines courses exceeded the recommended duration. Topical treatment prescription co-occurring with systemic antibiotics was surprisingly rare in both GPs and dermatologists, contrary to National Institute for Health and Care Excellence (NICE), European, and American guidelines.^8,9,31^

### Strengths and limitations

The data from several nationwide registries granting a large amount of patient and prescriber data is a major strength in our study, and it is the first to present nationwide data on GP prescription. Limitations include the lack of data on hormonal and spironolactone treatment, acne severity, and over-the-counter topical treatment. Spironolactone is, however, not approved for treatment of acne in Norway. Compliance with prescribed medications is also unknown and could be substantial.

### Comparison with existing literature

To our knowledge, no other study presents nationwide data on the prescription of acne medication by specialty. Buckley and Yoganathan described a case series where 100 patients received isotretinoin initiated by one GP, with extra interest and training in dermatology.^30,32^ The dermatological experience of the GPs in our study is unknown and likely varies, and the GP-initiated isotretinoin may have been prescribed after conferring with a local dermatologist. Increased initiation of isotretinoin therapy by GPs may reflect raised knowledge among GPs, limited dermatologists accessibility, growing patient demand, or lower threshold for patients to visit their GP. Similar patterns have been noted in other countries where GPs can prescribe isotretinoin.^29,30^ Our study indicates that 19% of GPs in Norway initiate isotretinoin, similar to the reported 17% from Ireland.^30^

Longer than recommended duration of antibiotic treatment has been reported in several studies, showing durations of 175 days in a UK study^16^ and 129 days in a systematic review,^13^ compared with 160 days in our study. Possible explanations for not following recommendations include long referral times to dermatologists, patient pressure for effective treatments, or lack of experience prescribing isotretinoin.

Odsbu *et al* observed a slight decrease in use of tetracyclines in Norwegian men from 2012 and women from 2014, until the end of their study in 2015.^17^ In our study, prevalence of tetracycline prescription continued to decrease in males but increased in females. This sex difference may be owing to a lower threshold for starting isotretinoin in males, as teratogenic side effects are not a concern. The overall increased use of isotretinoin may be owing to more prescribers having gained experience with its use and effectiveness or owing to efforts to reduce the use of antibiotics.

Prevalence of acne treatment prescription was inversely related to age for all subgroups except it was less common to prescribe isotretinoin and tetracycline to females aged 12–18 years compared with those aged 19–25 years. This is expected, as isotretinoin treatment requires prescription and use of contraceptives, which can be sensitive for the 12–15 age group, given that the legal age of sexual consent in Norway is 16 years.

Concurrent prescription of topical treatment together with systemic tetracyclines was low in our study, only 26%. Francis *et al* found that 34% of those receiving acne-related medication received oral antibiotics as monotherapy, and 7.5% had a combination of oral antibiotics and topical non-antibiotic treatments.^33^ In our study, over-the-counter topical treatment was not accounted for, nor if there were more than 90 days between the prescriptions, leaving the possibility that the numbers of actual concurrent treatment may be higher.

### Implications for research and practice

Increased GP prescribing and initiation of isotretinoin has potential to both reduce antibiotic prescription and to improve availability for optimal treatment for moderate-to-severe acne. However, the safety of GPs’ initiation remains to be investigated, specifically adherence to recommended follow-up for depression and contraception.

In conclusion, GPs decreased their prescription of tetracyclines but increased prescription of isotretinoin during the study period. Increased initiation of isotretinoin by GPs may be warranted, leading to improved treatment regimens, and decreasing antibiotic use. Safety of GP prescription remains to be addressed in further studies.

## Data

The underlying data for this article can be obtained from Norwegian authorities pending ethical approval in Norway.
